# A Short Version of Carers' Quality of Life Questionnaire for Parkinsonism: Data from Progressive Supranuclear Palsy Network

**DOI:** 10.1002/mdc3.70389

**Published:** 2025-10-16

**Authors:** Arianna Cappiello, Giovanna Calandra‐Buonaura, Roberto Ceravolo, Saverio Coralli, Sofia Cuoco, Eleonora del Prete, Francesca di Biasio, Daniela Frosini, Vittorio Gualtieri, Davide Mascioli, Luisa Sambati, Tommaso Schirinzi, Alessandro Stefani, Elisa Umbertini, Marco Aiello, Matteo Costanzo, Rosa de Micco, Giovanni Fabbrini, Gaetano Failla, Chiara Longo, Maria Chiara Malaguti, Nicola Modugno, Enrica Olivola, Noemi Maria Carola Russo, Alessandro Tessitore, Andrea Ciammola, Carlo Colosimo, Anna de Rosa, Laura de Togni, Alessio di Fonzo, Vincenzo Moschella, Andrea Pilotto, Francesca Spagnolo, Roberta Zangaglia, Paolo Barone, Marina Picillo, the PSP‐NET study group, Maria Autuori, Anna Rosa Avallone, Ilaria Cani, Giannicola Carrozzo, Nicola Grotteschi, Maria Mancini, Clara Simonetta, Maria Francesca Tepedino, Henri Zenuni, Simone Aloisio, Ruggero Bacchin, Lorena Belli, Daniele Belvisi, Francesco Marchet, Sara Pietracupa, Sara Satolli, Maria Concetta Altavista, Marta Filidei, Giulia Lazzeri, Simone Malaspina, Alessando Padovani, Gabriele Riccio, Andrea Mirko Tedesco, Susanne Buechner, Laura Bonanni, Marianna Capecci, Maria Gabriella Ceravolo, Massimo Cincotta, Maria Francesca de Pandis, Raffaella di Giacopo, Lorenza Leonardi, Leonardo Lopiano, Fabrizio Stocchi, Laura Vacca, Sara Varanese, Maristella Piccininni, Maurizio Zibetti

**Affiliations:** ^1^ Center for Neurodegenerative diseases (CEMAND), Department of Medicine, Surgery and Dentistry “Scuola Medica Salernitana” University of Salerno Salerno Italy; ^2^ Department of Biomedical and NeuroMotor Sciences Alma Mater Studiorum–University of Bologna Bologna Italy; ^3^ IRCCS Institute of Neurological Sciences Bologna Italy; ^4^ Neurology Unit, Department of Clinical and Experimental Medicine University of Pisa Pisa Italy; ^5^ Department of Neuroscience, Rehabilitation, Ophthalmology, Genetics, Maternal and Child Health (DINOGMI) University of Genoa Genoa Italy; ^6^ IRCCS San Martino General Hospital Genoa Italy; ^7^ Neurology Unit, Department of Systems Medicine Tor Vergata University of Rome Rome Italy; ^8^ IRCCS SYNLAB SDN Naples Italy; ^9^ Department of Human Neuroscience University of Rome Sapienza Rome Italy; ^10^ Department of Neuroscience National Institute of Health Rome Italy; ^11^ Department of advanced medical and surgical sciences University of Campania Luigi Vanvitelli Caserta Italy; ^12^ Neurology Unit, IRCCS Neuromed Pozzilli Italy; ^13^ Clinic for Movement Disorders Cannizzaro Hospital Catania Italy; ^14^ Department of Neurology Santa Chiara Hospital Trento Italy; ^15^ Department of Neurology and Laboratory of Neuroscience IRCCS Istituto Auxologico Italiano Milan Italy; ^16^ Dipartiment of Neurology Santa Maria University Hospital Terni Italy; ^17^ Neurology Unit Federico II University Naples Italy; ^18^ Complex Operative Unit of Neurology Magalini Hospital Verona Italy; ^19^ IRCCS Ca' Granda Ospedale Maggiore Policlinico, Neurology Unit Milan Italy; ^20^ Neurology Unit, San Filippo Neri Hospital ASL Roma1 Rome Italy; ^21^ Department of Clinical and Experimental Sciences University of Brescia Brescia Italy; ^22^ Department of Continuity of Care and Frailty, Unit of Neurology ASST Spedali Civili Brescia Italy; ^23^ Laboratory of Digital Neurology and Biosensors University of Brescia Brescia Italy; ^24^ Department of Neurology Antonio Perrino Hospital Brindisi Italy; ^25^ Parkinson's Disease and Movement Disorders Unit IRCCS Mondino Foundation Pavia Italy

**Keywords:** motor symptoms, neuropsychiatric symptoms, progressive supranuclear palsy

## Abstract

**Background and objectives:**

Caregivers of progressive supranuclear palsy (PSP) patients frequently show significant distress. The Parkinsonism Carers quality of life (QoL) (PQoL Carer) is a valid tool evaluating the effect of PSP on caregivers' QoL. Main aim of the present study was to develop a short version of the PQoL Carer, named PSP‐ShoQoL Carer.

**Methods:**

PQoL Carer was administered within the PSP‐NET. Participants underwent clinical, motor, cognitive, and behavioral evaluations.

**Results:**

Data from 344 participants were included. The final PSP‐ShoQoL Carer included eight items. The internal consistency was high (Cronbach's α = 0.867) and PSP‐ShoQoL Carer showed also good acceptability, reliability, and validity. The PSP‐ShoQoL Carer showed a significant correlation with caregivers' standard measures of QoL and with patients' motor, cognitive, and behavioral characteristics, such as neuropsychiatric symptoms. Finally, PSP‐ShoQoL Carer showed an appropriate sensitivity to change over 6‐month follow up.

**Conclusions:**

PSP‐ShoQoL Carer is a reliable and valid time‐saving tool for the assessment of caregivers' QoL in PSP.

Progressive supranuclear palsy (PSP) is a rare, atypical parkinsonism clinically defined by the Movement Disorder Society (MDS) diagnostic criteria.^1^, ^2^, ^3^ The rapid progression of the disease and the lack of effective treatments result in a considerable disability with a significant impact not only on patients' health‐related quality of life (QoL)^4^ but also on PSP caregivers' one.^5^, ^6^

The Parkinsonism Carers QoL (PQoL Carer) is a self‐administered questionnaire evaluating QoL of caregivers of patients with atypical parkinsonism.^7^ Although both the English and Italian versions have adequate psychometric properties,^8^ the PQoL Carer is time‐consuming for caregivers (~30–45 minutes).

Recently, a short version of the PSP QoL questionnaire (PSP‐QoL) has been developed.^9^

Hence, main aim of the present study was to develop a short version of the PQoL Carer, named PSP‐ShoQoL Carer, to ease QoL assessment in PSP patients' caregivers.

## Patients and Methods

Data derived from a cross‐sectional analysis of the PSP‐NET study and they were collected from both the patients and the caregivers (data downloaded on September 23, 2024).^10^ The project was performed in accordance with the ethical standards laid down in the 1964 Declaration of Helsinki.

The PQoL Carer was used to evaluate the quality of life of caregivers of PSP patients.^8^ Furthermore, caregivers also filled the following scales: (1) the three‐level version of the EuroQol scale (EQ‐5D) and the EQ‐visual analogue scale (EQ‐VAS)^11^; (2) the Hospital Anxiety and Depression Scale (HADS)^12^; (3) the Resilience Scale 14 (RS‐14)^13^; and (4) the Zarit burden interview (ZBI).^14^

Patients' motor burden was assessed with the Progressive Supranuclear Palsy‐rating scale (PSP‐rs).^15^, ^16^ Global cognition was evaluated with the Montreal Cognitive Assessment (MoCA).^17^ QoL was evaluated with the PSP‐QoL,^18^ the EQ‐5D,^11^ and the EQ‐VAS.^11^ Anxiety and depression were assessed with the HADS.^12^

Behavioral symptoms were evaluated with the Neuropsychiatric Inventory (NPI) and the Frontal Behavioral inventory (FBI), both filled by the caregiver.^19^, ^20^ Further details on Methods are available in Supporting information online (Table S1).

## Statistical Analysis

No subject had ≥30% of the data missing. For all other subjects, missing values were replaced using multiple imputation. Referring to the Markov Chain Monte Carlo (MCMC) method, 10 imputations were created in each case using 100 iterations.

To reduce the number of items, two criteria were applied. First, the factor loading on the associated factor should be high (here: above 0.60). Second, the corrected item‐scale correlations should be high (above 0.30). For the first criterion, we conducted a principal factor analysis of all 26 items of the scale with Varimax rotation. For the second one, we selected the items with the highest corrected item‐scale correlations. By this, eight items were selected. The total score, generating by summing items, ranges from 0 to 32 with higher values indicating a lower QoL.

Afterward, the following psychometric properties were explored for the PSP‐ShoQoL Carer total score: acceptability, reliability, and construct validity. Precision was evaluated by computing the standard error of measurement (SEM).

To characterize the relationship between PSP‐ShoQoL Carer and QoL, motor and cognitive symptoms measured with PSP‐QoL, PSP‐rs, and MoCA, a multiple linear regression analysis was conducted with PSP‐ShoQoL Carer as dependent variable and each clinical aspect as independent ones.

Finally, a test–retest analysis was used to analyze the sensitivity to change between baseline (T0) and 6‐month follow‐up (T1), using follow up data of a subsample of 60 caregivers.

## Results

A total of 344 caregivers of 344 PSP patients were included in the present study. Subjects' demographic and clinical features were displayed in Table 1. All 26 items were deemed appropriate for factorial analysis because Kaiser‐Meyer‐Olkin was 0.959. In addition, Bartlett's test was 5451.080 (*P* < 0.001) indicating that the data satisfied the condition of factorial analysis.

**TABLE 1:** Demographic and clinical features of the enrolled subjects

Caregivers	
Sex, male, n (%)	113 (32.8)
Age, y	61.08 ± 12.53
Hours spent with the patient, n (%)	
<12 h	99 (28.7)
≥12 h	245 (71.2)
Living with the patient, n (%)	
Yes	84 (24.4)
No	260 (75.5)
Relationship, n (%)	
Family member	289 (84)
Other	55 (15.9)
Center's locations, n (%)	
Northern Italy	144 (42)
Central Italy	107 (31.1)
Southern Italy	93 (27.03)
HADS, total score	11.28 ± 7.71
HADS, anxiety subscore	5.89 ± 4.27
HADS, depression subscore	5.39 ± 4.16
RS‐14	73.78 ± 13.48
ZBI	31.17 ± 15.11
**PSP patients**	
Sex, male, n (%)	185 (53.8)
PSP‐RS, n %	289 (84)
vPSP, n %	43 (12.5)
Age, y	72.15 ± 6.67
Disease duration, y	4.58 ± 2.79
Education, y	10.03 ± 4.16
PSP‐rs	42.82 ± 17.14
MoCA	17.14 ± 5.95
HADS, total score	16.81 ± 7.35
HADS, anxiety subscore	7.11 ± 3.79
HADS, depression subscore	9.92 ± 4.29
PSP‐QoL	79.63 ± 35.60

*Note*: Data are in mean ± standard deviation, unless otherwise specified. vPSP patients included PSP with predominant parkinsonism (PSP‐P = 19), PSP with predominant frontal presentation (PSP‐F = 4), PSP with progressive gait freezing (PSP‐PGF = 8), PSP with predominant ocular motor dysfunction (PSP‐OM = 3), PSP with speech/language disorder (PSP‐SL = 2), PSP with predominant postural instability (PSP‐PI = 4), PSP with predominant corticobasal syndrome (PSP‐CBS = 3). The phenotype for 12 patients was not specified.

Abbreviations: HADS, Hospital Anxiety And Depression Scale; RS‐14, Resilience Scale‐14; ZBI, Zarit burden Inventory; PSP, progressive supranuclear palsy; PSP‐RS, progressive supranuclear palsy with Richardson's syndrome; vPSP, other variant syndromes of PSP; PSP‐rs, Progressive Supranuclear Palsy‐rating scale; MoCA, Montreal Cognitive Assessment; PSP‐QoL, Progressive Supranuclear Palsy–Quality Of Life.

The factor analysis disclosed three factors (social environment, physical health, and caregiver–patient relationship strain), confirmed by the visual inspection of the screen plot. The developed three‐factor solution of the principal analysis of all 26 items explained 58.61% of total variance of our data. The criteria of the highest loading factors (>0.65) allowed the identification of 10 items. The items with corrected item‐scale correlations <0.30 were excluded. The final version of the PSP ShoQoL Carer results in eight items (Tables S2 and S3).

One hundred percent of data were totally computable and there were no missing values (0%). The mean (± standard deviation [SD]) PSP‐ShoQoL Carer total score was 12.76 ± 7.24. The item 6 (ie, do you go out less?, mean = 1.89) presented the highest score while the item 7 (do you find life boring?, mean = 0.98) displayed the lowest one. In the whole sample, neither ceiling nor floor effect were observed for the PSP‐ShoQoL Carer total score (lowest possible score = 0, 1.5%; highest possible score = 32, 0.6%). The skewness of the PSP‐ShoQoL Carer total score was 0.48 (criterion: −1 to +1).

Cronbach's α was 0.867 and, therefore, it was considered acceptable for internal consistency. No improvement of this value was noted on removal of any item.

Internal validity was confirmed by the significant positive correlation of the PSP‐ShoQoL Carer total score with all its items (*r* = 0.623, *P* < 0.001). The SEM value for PSP‐ShoQoL Carer total score was 0.390 (SEM = SD √ [1 – Cronbach's α]). The PSP‐ShoQoL Carer total score showed convergent validity with PSP QoL Carer total score, EQ‐5D Index, and its sub‐categories but it did not show correlation with EQ‐5D self‐care dimension, using for divergent validity (Table 2).

**TABLE 2:** Convergent and divergent validity of the PSP‐ShoQoL Carer total score

	*R*	*P*
PQoL caregiver	0.955	**<0.001**
EQ‐5D Index	−0.224	**<0.001**
EQ‐5D mobility	0.115	**0.050**
EQ‐5D self‐care	0.103	0.079
EQ‐5D usual activities	0.229	**<0.001**
EQ‐5D pain/discomfort	0.312	**<0.001**
EQ‐5D anxiety/depression	0.406	**<0.001**

*Note*: Bold‐typed values represent statistically significant findings.

Abbreviations: PSP‐ShoQoL Carer, short version of the Parkinsonism Carers quality of life; PQoL, Progressive Supranuclear Palsy Quality of Life; EQ‐5D, EuroQol.

Supporting the relationship between carer's behavioral symptoms and resilience and QoL, Pearson's correlation indicated a statistically significant correlation between the PSP‐ShoQoL Carer and the HADS caregiver total score (*r* = 0.457, *P* < 0.001), anxiety (*r* = 0.409, *P* < 0.001), and depression (*r* = 0.428, *P* < 0.001) scores, the RS‐14 (*r* = −0.205, *P* < 0.001), and the ZBI (*r* = 0.616, *P* < 0.001).

Confirming the impact of the patient's motor and behavioral disturbances on carer's QoL, PSP‐ShoQoL Carer showed a significant correlation with PSP‐rs (*r* = 0.399, *P* < 0.001), MoCA (*r* = −0.185, *P* = 0.001), HADS patients both total score (*r* = 0.306, *P* < 0.001), and anxiety (*r* = 0.209, *P* < 0.00) and depression (*r* = 0.305, *P* < 0.001) ones, PSP‐QoL patients total score (*r* = 0.477, *P* < 0.001), and motor (*r* = 0.413, *P* < 0.001) and cognitive (*r* = 427, *P* < 0.001) sub‐scores, EQ 5D Index (*r* = −0.300, *P* < 0.001), EQ 5D VAS (*r* = −.183, *P* < 0.001). Moreover, PSP‐ShoQoL Carer presented a significant positive correlation with the NPI total score (*r* = 0.307, *P* < 0.001) and the NPI Caregiver Distress (*r* = 0.316, *P* < 0.001) (Fig. S1) as well as with the FBI (*r* = 0.329, *P* < 0.001).

Multiple linear regression analysis identified the PSP‐QoL patient total score and the NPI total score as significant predictors of the PSP‐ShoQoL Carer showing a major impact of patient's QoL and behavioral disturbances on carer's QoL (PSP‐QoL patient total score: B = 0.077, *R* ^2^ adjusted = 0.264, *P* < 0.001; NPI total score: B = 0.097, *R* ^2^ adjusted = 0.264, *P* < 0.001).

A *t* test for paired samples revealed a significant increase of scores between T0 and T1, confirming the sensitivity to the expected changes of the PSP‐ShoQoL Carer (mean ± SD PSP‐ShoQoL Carer total score T0 12.76 ± 7.24; mean ± SD PSP‐ShoQoL Carer total score T1 13.62 ± 7.20, *P* < 0.01) after 6 months from baseline assessment. Moreover, test re‐test reliability was acceptable (*r* = 0.54, *P* < 0.001), meaning that the PSP‐ShoQoL Carer measures inter individual differences.

## Discussion

Main objective of the present study was to develop a short version of the PQoL Carer.

Our factorial analysis showed the PSP‐ShoQoL Carer includes three factors representing functional and psychological aspects of daily living. They can be named as follows: (1) social environment; (2) physical health; and (3) caregiver–patient relationship strain.^21^, ^22^ The first factor includes social withdrawal, boredom, and social isolation and reflect the sacrifice of caregivers who renounce to their hobbies and time spent with friends, family, and at work.^23^ Physical stress and fatigue resulting from caregiving were related to the second factor labeled physical health.^24^ Finally, the caregiver‐patient relationship strain factor covers communication changes, personality and role transformations.^25^, ^26^

As regards psychometric properties, the PSP‐ShoQoL Carer showed high acceptability, supported by the absence of both ceiling and floor effects. The internal consistency of the PSP‐ShoQoL Carer was high and acceptable (α = 0.867). The degree of precision of measurement, expressed in terms of SEM, was adequate suggesting the PSP‐ShoQoL Carer values in our sample were an accurate estimation of the PSP‐ShoQoL Carer values in the caregiver population.

The PSP‐ShoQoL Carer showed a significant association with EQ‐5D Index and with mobility, usual activities, pain/discomfort,anxiety/depression domains of EQ‐5D, while it was not correlated with self‐care one. This result was in accordance with our assumptions because both PSP‐ShoQoL Carer, EQ‐5D Index, and physical and psychological domains measured with the EQ‐5D measured the same aspects of daily life. Previous studies showed that neurodegenerative diseases' caregivers presented an impairment of QoL.^27^, ^28^ The lack of correlation between the PSP‐ShoQoL Carer and self‐care domain of EQ‐5D revealed divergent construct validity (ie, a measure does not correlate strongly with measures of different, unrelated constructs), as this construct was measured by EQ‐5D, but not by the PSP‐ShoQoL Carer.

We also observed a correlation between the PSP ShoQoL Carer total score and clinical characteristics, measured with HADS and ZBI. In line with a previous study, PSP caregivers with worse affective symptoms, like depression and anxiety, and burnout presented a greater functional impact from PSP.^29^

Moreover, resilience, measured by RS‐14, presented an inverse correlation with caregiver QoL. Therefore, caregivers with reduced resilience are more prone to have a reduced QoL as consequence of caregiving.^30^

PSP‐ShoQoL Carer also showed significant correlations with PSP patients' characteristics. In line with previous findings, caregivers of PSP patients presenting a worse motor and cognitive disturbance, exhibited a worse QoL.^20^ Both positive and negative PSP neuropsychiatric symptoms constituted a further source of caregiver distress. In line with previous evidence reported for Parkinson's disease,^31^ we showed that PSP‐ShoQoL Carer is related to both NPI total score and NPI Caregiver Distress. As regards the sensibility to change we demonstrated the PSP‐ShoQoL Carer reliably detects the expected changes over time.

We acknowledge our study has same limitations. First, our cohort was mostly represented by caregivers of PSP‐RS patients. Moreover, most caregivers (84.01%) were family members. Finally, validation of the PSP‐ShoQoL Carer in an independent cohort would be advisable.

In conclusion, the PSP‐ShoQoL Carer provides a brief and more focused assessment showing high acceptability, validity and reliability in assessing QoL in PSP caregivers.

## Author Roles

(1) Research project: A. Conception, B. Organization, C. Execution; (2) Statistical analysis: A. Design, B. Execution, C. Review and critique; (3) Manuscript: A. Writing of the First Draft, B. Review and critique.

A.C.: 1C, 2A, 2B, 2C, 3A, 3B

G.C.B.: 2B, 3B

R.C.: 2B, 3B

S.V.: 2B, 3B

S.C.: 1C, 2A, 2B, 2C, 3A

E.D.P.: 2B, 3B

F.D.B.: 2B, 3B

D.F.: 2B, 3B

V.G.: 2B, 3B

D.M.: 2B, 3B

L.S.: 2B, 3B

T.S.: 2B, 3B

A.S.: 2B, 3B

E.U.: 2B, 3B

M.A.: 2B, 3B

M.C.: 2B, 3B

R.D.M.: 2B, 3B

G. Fabbrini: 2B, 3B

G. Failla: 2B, 3B

C.L.: 2B, 3B

M.C.M.: 2B, 3B

N.M.: 2B, 3B

E.O.: 2B, 3B

N.M.C.R.: 2B, 3B

A.T.: 2B, 3B

A.C.: 2B, 3B

C.C.: 2B, 3B

A.D.R.: 2B, 3B

L.D.T.: 2B, 3B

A.D.F.: 2B, 3B

V.M.: 2B, 3B

A.P.: 2B, 3B

F.S.: 2B, 3B

R.Z.: 2B, 3B

P.B.: 2B, 3B

M.P.: 1A, 1B, 2C, 3B

## Disclosures

**Ethical Compliance Statement:** We confirm that we have read the Journal's position on issues involved in ethical publication and affirm that this work is consistent with those guidelines. The study protocol was approved by the local ethics committee (approval number 32 on December 2, 2020, Comitato etico Campania Sud) and an informed consent was signed by participants before recruitment. The authors have no conflict interests to declare that are relevant to the content of this article. An informed consent was signed by participants before recruitment.

**Funding Sources and Conflict of Interest:** The Fondazione LIMPE per il Parkinson Onlus supported this project. The authors declare no potential conflict of interest or financial disclosure related to the material in the article.

**Financial Disclosures for the Previous 12 Months:** M.P. is supported by the Italian Ministry of Health, the Italian Ministry of University and the Fondazione della Società Italiana di Neurologia. P.B. received consultancies as a member of the advisory board for Zambon, Lundbeck, UCB, Chiesi, AbbVie, and Acorda. The other authors declare that there are no additional disclosures to report.

## Supporting information


**Figure S1.** reported the neuropsychiatric and behavioralbehavioural correlations of PSP‐ShoQoL Carer. PSP‐ShoQoL Carer presented a significant positive correlation with the NPI total score (NPI FxS), given by the sum of the product of frequency and severity, and the NPI Caregiver Distress.
**TABLE S1.** reported further details on Methods.
**TABLE S2.** reported the English version of the short version of the PSP‐QoL Carer (PSP‐ShoQoL Carer).
**TABLE S3.** reported the Italian version of the short version of the PSP‐QoL Carer (PSP‐ShoQoL Carer).

## Data Availability

Anonymized data, statistical methods, and experimental material not entirely published within the article will be shared by request to the corresponding author from any qualified investigator.

## Appendix

**Members of the PSP‐NET study group**

Maria Autuori^1^, Anna Rosa Avallone^1^, MD, Ilaria Cani^2^, MD, PhD, Giannicola Carrozzo^3^, MD, Nicola Grotteschi^3^, MD, Maria Mancini^4^, MD, Clara Simonetta^4^, MD, PhD, Maria Francesca Tepedino^1^, MD, PhD, Henri Zenuni^4^, MD, Simone Aloisio^5^, MD, PhD, Ruggero Bacchin^6^, MD, Lorena Belli^7^, MD, PhD, Daniele Belvisi^7,8^, MD, PhD, Francesco Marchet ^8^, MD, Sara Pietracupa^7^, MD, PhD, Sara Satolli^5^, MD, Maria Concetta Altavista^9^, MD, PhD, Marta Filidei^10^, MD, Giulia Lazzeri^11^, MD, Simone Malaspina^12 13^, MD, Alessando Padovani^14,15,16^, MD, PhD, Gabriele Riccio^17^, MD, Andrea Mirko Tedesco^18^, Susanne Buechner^19^, MD, Laura Bonanni^20^, MD, PhD, Marianna Capecci^21^, MD, PhD, Maria Gabriella Ceravolo^21^, MD, PhD, Massimo Cincotta^22^, MD, Maria Francesca de Pandis^23,24^, MD, PhD, Raffaella Di Giacopo^25^, MD, PhD, Lorenza Leonardi^23^, Leonardo Lo Piano^26^, MD, PhD, Fabrizio Stocchi^27^, MD, PhD, Laura Vacca^27^, MD, PhD, Sara Varanese^20^, MD, PhD, Maristella Piccininni^22^, MD, Maurizio Zibetti^26^, MD, PhD.

**Affiliations of the PSP‐NET study group.**

^1^Center for Neurodegenerative diseases (CEMAND), Department of Medicine, Surgery and Dentistry “Scuola Medica Salernitana”, University of Salerno, Salerno, Italy.

^2^IRCCS, Istituto delle Scienze Neurologiche di Bologna, Italia.

^3^Dipartimento di Scienze Biomediche e Neuromotorie, Alma Mater Studiorum – Università di Bologna, Italia.

^4^Neurology Unit, Department of Systems Medicine, Tor Vergata University of Rome, Rome, Italy.

^5^Department of advanced medical and surgical sciences, University of Campania Luigi Vanvitelli, Caserta, Italy.

^6^Department of Neurology, Santa Chiara Hospital, Trento, Italy.

^7^Neurology Unit, IRCCS Neuromed, Pozzilli, IS, Italy.

^8^Department of Human Neuroscience, University of Rome Sapienza, Rome, Italy.

^9^Neurology Unit, San Filippo Neri Hospital ASL Roma1, Rome, Italy.

^10^Dipartiment of Neurology, Santa Maria University Hospital, Terni, Italy.

^11^IRCCS Ca′ Granda Ospedale Maggiore Policlinico, Neurology Unit, Milan, Italy.

^12^Parkinson's Disease and Movement Disorders Unit, IRCCS Mondino Foundation, Pavia, Italy.

^13^Department of Brain and Behavioral Sciences, University of Pavia, Pavia, Italy.

^14^Department of Clinical and Experimental Sciences, University of Brescia, Brescia, Italy.

^15^Department of Continuity of Care and Frailty, Unit of Neurology, ASST Spedali Civili, Brescia, Italy.

^16^Laboratory of Digital Neurology and Biosensors, University of Brescia, Brescia, Italy.

^17^Neurology Unit, Federico II University, Naples, Italy.

^18^Department of Neurology, Antonio Perrino's Hospital, Brindisi, Italy.

^19^Neurology Unit, Bolzano Hospital, Bolzano, Italy.

^20^Department of Neuroscience, Imaging and Clinical Sciences, University G. d'Annunzio of Chieti‐Pescara, Chieti, Italy.

^21^Neurorehabilitation Clinic, Ancona Hospital, Ancona, Italy.

^22^SOC of Neurology Florence, USL Tuscany‐Centre, Florence, Italy.

^23^San Raffaele Cassino, Cassino, Italy.

^24^Telematic University San Raffaele Rome, Rome, Italy.

^25^Neurology Unit, Santa Maria del Carmine Hospital, Rovereto (TN).

^26^Department of Neuroscience, University of Torino, Torino, Italy.

^27^IRCCS San Raffaele, Rome, Italy.
