# Supplementary material for: A Short Version of Carers' Quality of Life Questionnaire for Parkinsonism: Data from Progressive Supranuclear Palsy Network
Source: Mov Disord Clin Pract. 2025 Oct 16;13(4):1038–43. doi: 10.1002/mdc3.70389 (PMC13071305; doi:10.1002/mdc3.70389)
Supplement: Supplementary file 1 — Figure S1. reported the neuropsychiatric and behavioralbehavioural correlations of PSP‐ShoQoL Carer. PSP‐ShoQoL Carer presented a significant positive correlation with the NPI total score (NPI FxS), given by the sum of the product of frequency and severity, and the NPI Caregiver Distress. TABLE S1. reported further details on Methods. TABLE S2. reported the English version of the short version of the PSP‐QoL Carer (PSP‐ShoQoL Carer). TABLE S3. reported the Italian version of the short version of the PSP‐QoL Carer (PSP‐ShoQoL Carer). [file MDC3-13-1038-s001.docx]

**Supplemental Table 1. Further details on Methods**

| **Methods**  Caregivers filled the following scales: (1) the Parkinsonism Carers QoL (PQoL Carer); (2) The three-level version of the EuroQol scale (EQ-5D) and the EQ-visual analogue scale (EQ-VAS); (3) Hospital Anxiety and Depression Scale (HADS); (4) the Resilience Scale 14 (RS-14); (5) the Zarit burden interview (ZBI).  The PQoL Carer is a caregiver-oriented, 26-item questionnaire with each item scored on 5-point Likert scale (0 = none; 1 = mild; 2 = moderate; 3 = severe; 4= extreme). The PQoL Carer total score ranges from 0 to 78 with 62 as proposed cut-off indicating greater burden in PSP caregivers.  The EQ-5D is a 5-item self-report scale concerning the individual’s level of health status on five dimensions (mobility, self-care, usual activities, pain/discomfort, and anxiety/depression).  The EQ-VAS is a self-rating of overall health ranging from 0 (worst imaginable health state) to 100 (best imaginable health state).  The HADS is a 14 items scale encompassing both anxiety (7 items) and depression (7 items) symptoms each ranked on a 4-point Likert scale from 0 to 3 with the total score ranges from 0 to 21 for either anxiety and depression.  The RS-14 is constituted by 14 items requesting to declare the agreement on a 7-point Likert scale with a score < 56 indicates a very low level of resilience.  The ZBI measures the caregiver’s burnout thought a 22-items on a 5-point Likert scale (from 0 “Never” to 4 “Almost always”).  Moreover, behavioural symptoms were evaluated with the Neuropsychiatric Inventory (NPI) and the Frontal Behavioural inventory (FBI), both filled by the caregiver. The NPI investigates 12 different behavioural difficulties (delusions, hallucinations, agitation/aggression, depression/dysphoria, anxiety, elation/euphoria, apathy/indifference, disinhibition, irritability/lability, motor disturbance, night-time behaviours, appetite/eating disorders). For each symptom, caregiver reports both frequency (1= occasionally, less than once per week; 2= often, once a week; 3 = frequently, several times a week but not every day; 4= very frequently, once or more per day) and severity (1= mild; 2= moderate; 3= marked). The total score is given by the sum of the product of frequency and severity for each symptom and ranges from 0 to 144. Moreover, the scale includes an instrument for the evaluation of the caregiver distress determined by each symptom (0 = no distress; 1= minimal; 2= mild; 3= moderate; 4 = moderately severe; 5= very severe or extremely severe). The Frontal Behaviour Inventory (FBI) is a 24-item questionnaire divided into two sub-scores: FBI-A (items 1 to 12) that investigates negative behaviour (i.e., indifference, apathy, inflexibility, personal neglect) and FBI-B (items 13 to 24) that explores disinhibition symptoms (i.e., irritability, impulsivity, aggression, excessive jocularity), with each item scored on 4-point Likert scale (0 = none; 1 = mild or occasional; 2 = moderate; 3 = severe or most of the time). |
| --- |

**Supplemental Table 2. Short version of the PSP-QoL Carer (English version)**

| **In the previous 4 weeks** | Never | Mild difficulty | Moderate difficulty | Severe difficulty | Extreme difficulty | Not applicable |
| --- | --- | --- | --- | --- | --- | --- |
| 1. Do you find it a physical strain to look after your relative/partner? |  |  |  |  |  |  |
| 2. Do you feel fatigued or tired? |  |  |  |  |  |  |
| 3. Has your ability to communicate with your relative/partner suffered? |  |  |  |  |  |  |
| 4. Do you find it difficult to deal with your relative’s/partner’s changed personality? |  |  |  |  |  |  |
| 5. Do you find it difficult to deal with a change in roles between you and your relative or partner? |  |  |  |  |  |  |
| 6. Do you go out less? |  |  |  |  |  |  |
| 7. Do you find life boring? |  |  |  |  |  |  |
| 8. Do you see friends and family less? |  |  |  |  |  |  |

Please verify that you have marked X in only one box for each question.

Having any illness affects your personal life.

Please indicate how satisfied you feel with your life in general right now by marking an X on a point on the scale between 0 and 100.


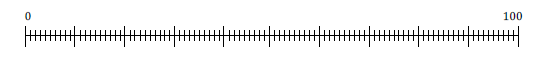


Extremely dissatisfied Extremely satisfied

of my life of my life

Did you complete the questionnaire yourself? □

Do you have any other comments?

**Thank you for completing the questionnaire!**

**Supplemental Table 3. Short version of the PSP-QoL Carer (Italian version)**

Assistere una persona con un problema di salute può influire sulla qualità di vita di una persona in molti modi diversi. Per aiutarci a capire come la malattia della persona che assiste influisce sulla sua vita, vorremmo sapere se Lei ha mai presentato le seguenti difficoltà e quanto ognuna è stata problematica. Se qualcuna delle difficoltà non La riguarda, per favore ne annoti il motivo. Se qualcuno La aiuta a riempire il questionario, per piacere si assicuri che le risposte riflettano il Suo personale punto di vista. Per favore tenga presente che questa lista include molte difficoltà che forse Lei non ha mai presentato.

Non ci sono risposte giuste o sbagliate e vorremmo che Lei:

• pensi a come si è sentito nelle scorse quattro settimane

• segnare una X nella casella corrispondente alla risposta che meglio rappresenta la sua situazione

| **Nelle precedenti 4 settimane** | Mai | Difficoltà lieve | Difficoltà moderata | Difficoltà grave | Difficoltà estrema | Non applicabile |
| --- | --- | --- | --- | --- | --- | --- |
| 1. Prendersi cura del Suo parente/partner necessita di uno sforzo fisico? |  |  |  |  |  |  |
| 2. Si sente affaticato o stanco? |  |  |  |  |  |  |
| 3. La comunicazione con il Suo parente/partner è peggiorata? |  |  |  |  |  |  |
| 4. Pensa sia difficile avere a che fare con il cambio di personalità del suo parente/partner? |  |  |  |  |  |  |
| 5. Pensa sia difficile tollerare il cambio di ruoli tra Lei e il Suo parente/partner? |  |  |  |  |  |  |
| 6. Esce di meno? |  |  |  |  |  |  |
| 7. Trova che la vita sia noiosa? |  |  |  |  |  |  |
| 8. Vede meno amici e familiari? |  |  |  |  |  |  |

Per favore verifichi di aver segnato la X in una sola casella per ciascuna domanda.

Avere una qualsiasi malattia ha conseguenze sulla vita personale.

Per favore indichi quanto si sente soddisfatto della sua vita in generale in questo momento segnando una X su un punto della scala tra 0 e 100.


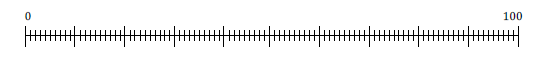


Estremamente insoddisfatto Estremamente soddisfatto

della mia vita della mia vita

Ha completato il questionario da solo? □

Ha altri commenti?

**Grazie per aver completato il questionario!**

**Supplemental Figure 1. Neuropsychiatric and behavioural correlates of PSP-ShoQoL Carer**


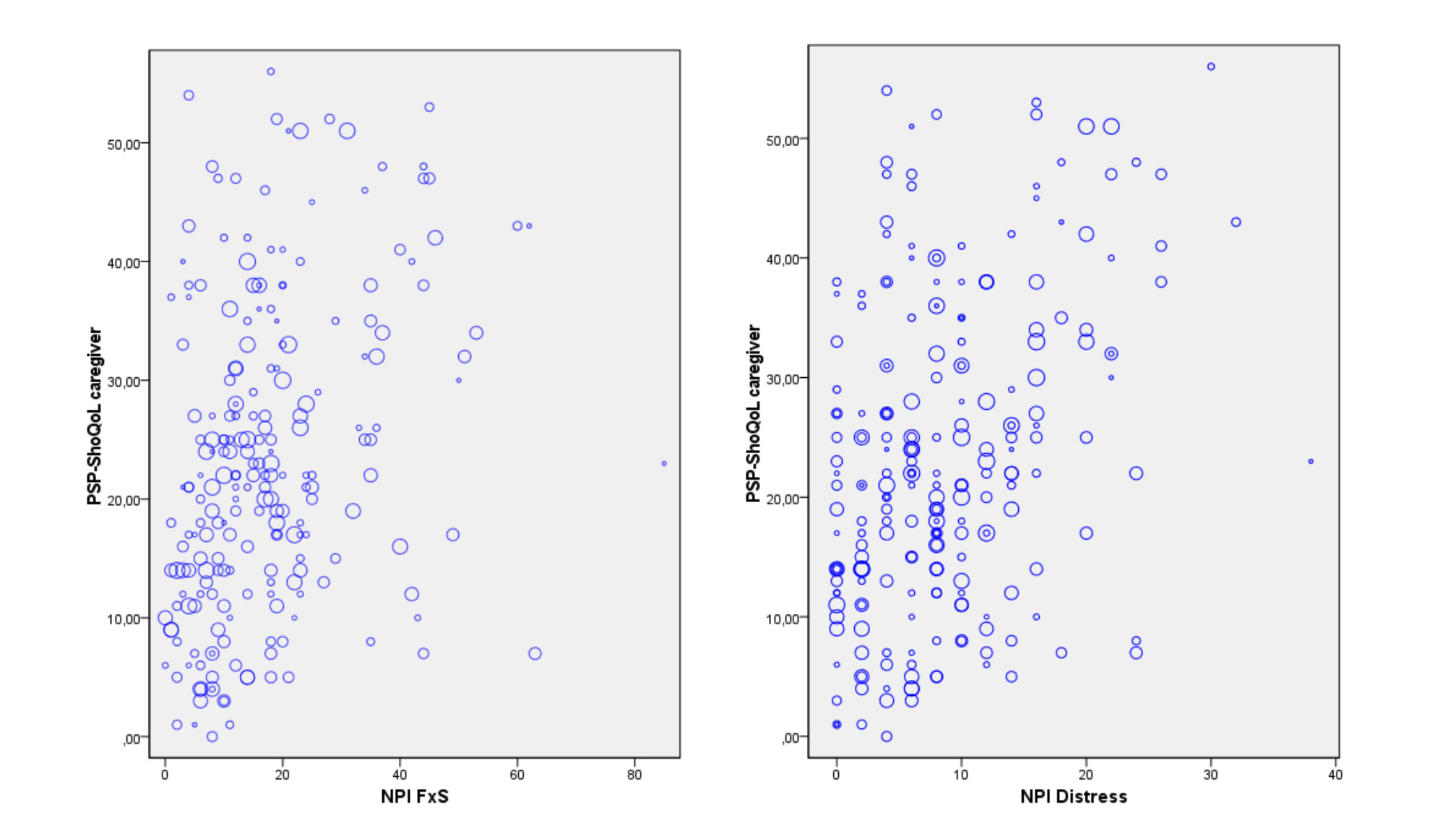


B figure has multiple panels,

A figure has multiple panels,

Abbreviations: PSP-ShoQoL, Progressive Supranuclear Palsy quality of life short version; NPI, Neuropsychiatric Inventory; FxS, the product of frequency and severity.
